# Maheshvara regulates JAK/STAT signaling by interacting and stabilizing *hopscotch* transcripts which leads to apoptosis in *Drosophila melanogaster*

**DOI:** 10.1038/s41419-021-03649-0

**Published:** 2021-04-06

**Authors:** Bhawana Maurya, Satya Surabhi, Rituparna Das, Pranjali Pandey, Ashim Mukherjee, Mousumi Mutsuddi

**Affiliations:** 1 Department of Molecular and Human Genetics, Institute of Science, Banaras Hindu University, Varanasi, 221005 Uttar Pradesh India; 2 Present Address: Department of Molecular and Developmental Biology, Albert Einstein College of Medicine, New York, NY 10461 USA

**Keywords:** Apoptosis, Cell signalling

## Abstract

Maheshvara (*mahe*), an RNA helicase that is widely conserved across taxa, regulates Notch signaling and neuronal development in *Drosophila*. In order to identify novel components regulated by *mahe*, transcriptome profiling of ectopic *mahe* was carried out and this revealed striking upregulation of JAK/STAT pathway components like *upd1*, *upd2*, *upd3*, and *socs36E*. Further, significant downregulation of the pathway components in *mahe* loss-of-function mutant as well as upon lowering the level of *mahe* by *RNAi*, supported and strengthened our transcriptome data. Parallelly, we observed that *mahe*, induced caspase-dependent apoptosis in photoreceptor neurons, and this phenotype was significantly modulated by JAK/STAT pathway components. RNA immunoprecipitation unveiled the presence of JAK/STAT tyrosine kinase *hopscotch* (*hop*) transcripts in the complex immunoprecipitated with Mahe, which ultimately resulted in stabilization and elevation of *hop* transcripts. Additionally, we also observed the surge in activity of downstream transcription factor Stat92E, which is indicative of activation of the JAK/STAT signaling, and this in turn led to apoptosis via upregulation of *hid*. Taken together, our data provide a novel regulation of JAK/STAT pathway by RNA helicase Maheshvara, which ultimately promotes apoptosis.

## Introduction

With the development of a deeper understanding of the importance of RNA in maintaining the fidelity of biological processes, the pivotal roles played by RNA binding proteins (RBPs)^1,2^ such as the DEAD box RNA helicases (DBRHs) are being unraveled. The DBRHs are a set of multitasking proteins that are involved in multitudes of RNA processes depending largely on the partners they interact with in various biological contexts^3,4^. The involvement of DBRHs in RNA processing events in neurons implicates their role in numerous neurological disorders^4,5^. High-throughput approaches have identified mutations in DEAD box helicase genes as major cause of neurodevelopmental disabilities, these include *DDX59*, *DDX6*, *DHX37*, *DHX30*, *DHX16*, *DHX34*, and *DDX54*^6–9^.

We have previously characterized *maheshvara (mahe)*, a *Drosophila* homolog of human *DDX59*, as a novel modulator of Notch signaling^10^. Misregulated *mahe* leads to neuronal defects and reduced lifespan in *Drosophila*, which parallels similarities with patients having frameshift deletion in *DDX59* presenting orofaciodigital syndrome associated with broad neurological defects^11^.

*mahe* and its human orthologues have been implicated in disease and development of nervous system. To delve deeper into the mechanism of the neuronal defects caused due to ectopic *mahe*, we carried out whole transcriptome analysis. Interestingly, we observed significant upregulation of JAK/STAT pathway components. In addition, alike to the case of many DEAD box helicase associated neurological diseases^12,13^, we also noted that overexpression of *mahe* leads to increased apoptosis in the developing photoreceptor neurons. *Drosophila* JAK/STAT signaling, though relatively simpler when compared to that of vertebrates, regulates patterning, organizer establishment, proliferation, apoptosis and maintains the size of the adult *Drosophila* eye^14^. Mechanistically, secreted ligand family unpaired (*upd*) and *upd* like proteins (*upd2* and *upd3*) binding dimerizes the receptor (*domeless*/*dome*) and activates the receptor-associated JAK tyrosine kinases (*hopscotch*/*hop*). In the canonical pathway, these tyrosine kinases phosphorylate themselves and their associated receptors and this creates docking sites for signal-transducer and activator of transcription protein at 92E (*Stat92E*). Stat92E transcription factor dimerizes upon phosphorylation and enters into the nucleus to activate or repress transcription of target genes such as *socs36E*^15,16^. During development, the decision of a cell to die or survive requires the integration of several regulatory pathways among which JAK/STAT signaling also plays a significant role. In our study, genetic analysis of *mahe* with members of JAK/STAT pathway displayed modulation of apoptotic phenotype in the developing photoreceptor neurons. Our study has uncovered the novel role of an RNA helicase Mahe that binds to *hop* tyrosine kinase transcripts, and leads to a surge in its levels resulting in activation of JAK/STAT signaling. This ectopic JAK/STAT activation culminates into *hid* mediated cell death. To the best of our knowledge, this is the first report in either vertebrates or invertebrates, where the pivotal role played by an RNA helicase in activating JAK/STAT cascade has been unraveled. This activation ultimately ensues in an upregulation of *hid* mediated apoptosis.

## Results

### Transcriptome analysis of *mahe* reveals upregulation of JAK/STAT pathway components

**Fig. 1:**
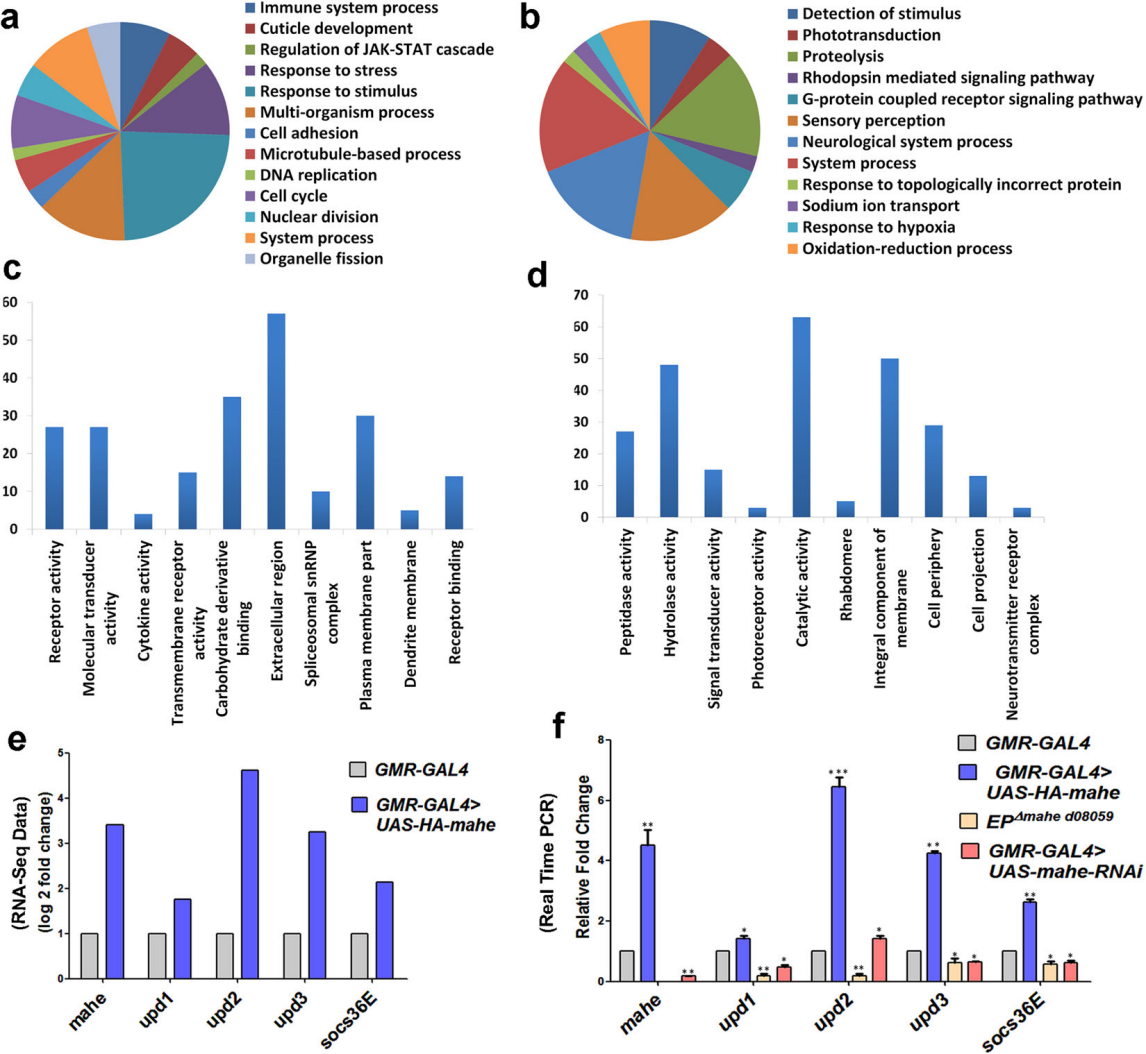
*mahe* promotes upregulation of members of JAK/STAT pathway as revealed by transcriptome analysis. Transcriptome analysis was done of eye tissue overexpressing *mahe* driven with *GMR-GAL4* and was compared to *GMR-GAL4* controls. **a**, **b** Functional categories enriched in upregulated (1080) and downregulated (519) genes based on their molecular functions using DAVID, which includes genes associated with phototransduction, JAK/STAT pathway activation, response to stress, nuclear division, and many more. **c**, **d** Enrichment of pathway specific upregulated (1080) and downregulated (519) genes based on their biological and cellular functions using DAVID. **e** Graph represents selected genes *upd1*, *upd2*, *upd3* and *socs36E* were differentially expressed in the RNA-Seq data. **f** Real time PCR validated the candidate genes obtained from our transcriptome analysis data. The components of JAK/STAT signaling pathway were upregulated upon ectopic expression of *mahe* and were significantly downregulated in *mahe* loss-of-function mutant as well as in *mahe-RNAi*. One-way ANOVA. Genotypes (**a**–**f**) *w/+; +; GMR-GAL4/*+ and *w/+; +; GMR-GAL4, UAS-HA-mahe/UAS-HA-mahe* (**f**) *EP*^*∆mahe d08059*^*;+;+* (**f**) +; *UAS-mahe-RNAi/+; GMR-GAL4/*+. One-way ANOVA *P < 0.05, **P < 0.01, ***P < 0.001.

For dissecting out the molecular factors regulated by *mahe* during development, transcriptome analysis using RNA extracted from *GMR-GAL4* driven *mahe* overexpressed eye tissues were compared to that of the transcript levels in *GMR-GAL4* controls. RNA-sequencing analysis revealed a total of 12,225 differentially regulated genes (with fold change > 0.5). Out of these, 1080 genes were found to be upregulated and 519 genes were downregulated significantly (with fold change >1.5). For functional annotation of differentially expressed genes mined from our RNA-Seq data, NIH DAVID 6.8 (The Database for Annotation, Visualization, and Integrated Discovery) was used. Gene Ontology (GO) analysis, identified the biological activities and molecular pathways affected in response to *mahe* overexpression (Fig. 1a–d).

Interestingly, DAVID analysis revealed positive regulation of JAK/STAT cascade (Fig. 1a). To reconfirm our RNA-Seq data, real-time PCR was done in *mahe* loss-of-function mutant, remarkably each of the three *Drosophila* JAK/STAT ligands *upd1*, *upd2*, *upd3*, and *socs36E*, the downstream target of the transcription factor Stat92E were significantly downregulated (Fig. 1f). As there were very few first instar escaper larvae of *mahe* loss-of-function mutant and none developed up to third instar larval stage, further analysis was done using *mahe-RNAi*. In addition, in line with that of *mahe* mutants, JAK/STAT pathway components were downregulated upon lowering the levels of *mahe* by *RNAi* (Fig. 1f). Further, in agreement with the above results ectopically expressed *mahe* tissue revealed upregulation of the JAK/STAT signaling components, and this corresponded with the RNA-Seq data (Fig. 1e, f). Together, these data indicated *mahe* to be a positive regulator of JAK/STAT pathway.

### *mahe* induces Caspase-dependent cell death in a dosage sensitive manner

**Fig. 2:**
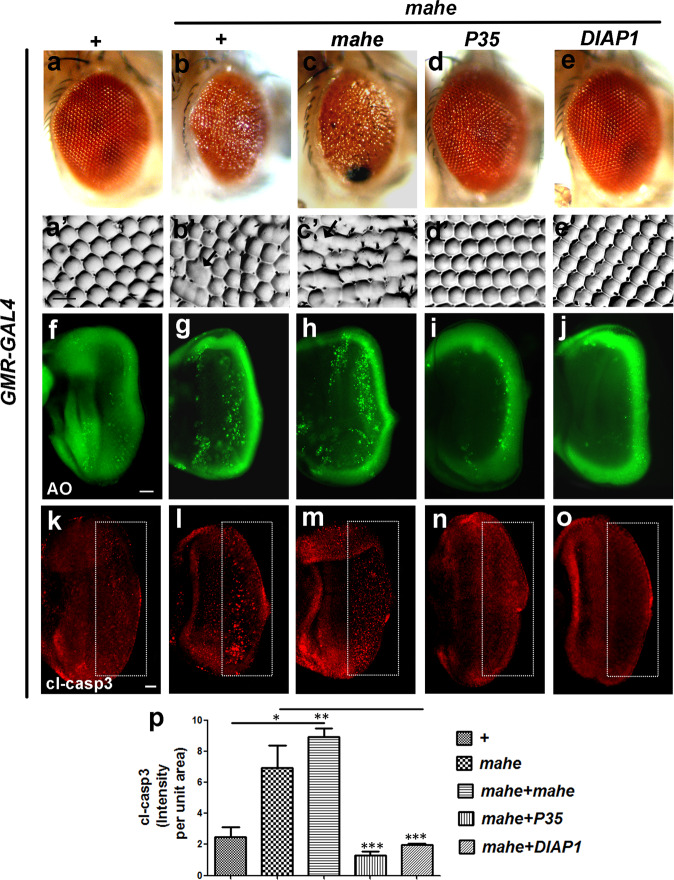
Ectopic expression of *mahe* promotes caspase-dependent dosage sensitive cell death in eye tissue of *Drosophila*. **a**–**e** Images of *Drosophila* adult eyes. **a′**–**e′** Eye imprints of adult eye. *GMR-GAL4* was used as a control (**a**), or to drive expression of *mahe* (**b**–**e**), *P35* (**d**), *DIAP1* (**e**). **a**, **a′** Control adult eye showed normal ommatidium arrangement. **b**, **b′** *GMR-GAL4* > *mahe* induced eye roughness with fused ommatidia (marked with arrow). **c**, **c′** Ommatidial disarray and fusion were severely enhanced on increasing the dosage of *mahe*. **d**, **d′** *mahe* induced eye phenotype was suppressed by expression of *P35*, an inhibitor of effector caspase. **e**, **e′** Similar to *P35*, inhibitor of initiator caspase *DIAP1* suppressed the eye roughening and ommatidium disarrangement. **f**–**j** Acridine orange staining of eye-antennal imaginal discs. **f** Acridine orange marks few dying cells in control eye-antennal disc. **g**, **h** Number of acridine orange positive cells were enhanced upon *mahe* overexpression in dosage sensitive manner. **i**, **j** *P35* and *DIAP1* suppressed cell death, shown by reduction in acridine positive nuclei. **k–o** Caspase immunostaining of eye-antennal discs from third instar larvae (Compare area within rectangle). **l**, **m** Ectopic *mahe* leads to increase in number of caspase positive cells in comparison to wild type (**k**). **n**, **o** Reduction in caspase positive cells were observed upon coexpression of *P35* and *DIAP1* along with *mahe*. (**p**) Graph represents intensity of cl-casp3 per unit area showing *mahe* triggers caspase-dependent cell death, which was suppressed by *P35* and *DIAP1*. One-way ANOVA. Scale bar in 50μm (**a′**–**e′**, **f**–**j**, **k**–**o**). Genotypes (**a**) *w/+; +; GMR-GAL4/*+ (**b**) *w/+; +; GMR-GAL4, UAS-HA-mahe/+* (**c**) *w/+; +; GMR-GAL4,UAS-HA-mahe/UAS-HA-mahe* (**d**) *w/+; +; GMR-GAL4,UAS-HA-mahe/UAS-P35* (**e**) *w/+; +; GMR-GAL4,UAS-HA-mahe/UAS-DIAP1*. One-way ANOVA * *P* < 0.05, ** *P* < 0.01, *** *P* < 0.001.

Parallelly, a gain-of-function assay was carried out and the effect of ectopic expression of *mahe* was monitored in the developing photoreceptors. An HA tagged full length Mahe protein was ectopically expressed posterior to the morphogenetic furrow using *GMR-GAL4* driver. Overexpression of *mahe* resulted in rough and reduced eye phenotype when compared to the *GMR-GAL4* control adult eye phenotype (Fig. 2b, b′ and c, c′ to a, a′). The rough eye phenotype was found to be largely due to apoptosis, as seen by acridine orange staining as well as immunostaining of cleaved caspase and the immunofluorescence signal was dosage sensitive to the levels of *mahe* expression (Fig. 2g, l and h, m to f, k). Cell survival is facilitated by factors which directly inhibit caspases. *DIAP1* and *P35* are such known inhibitors of caspases. Our observation was further consolidated by the significant rescue of *mahe* associated eye roughness and apoptosis by *DIAP1* and *P35* (Fig. 2d, d′, i, n and e, e′, j, o to b, b′, g, l). Quantitative analysis of cleaved caspase staining in terms of intensity per unit area was indicated by prominent reduction in apoptotic cells when *P35* and *DIAP1* are coexpressed with *mahe* (2 p). These results indicate that *mahe* induces cell death in canonical caspase-dependent manner and its level must be tightly regulated for proper development and differentiation of *Drosophila* eye.

### *mahe* induced apoptosis is mediated via surge in JAK/STAT pathway components

**Fig. 3:**
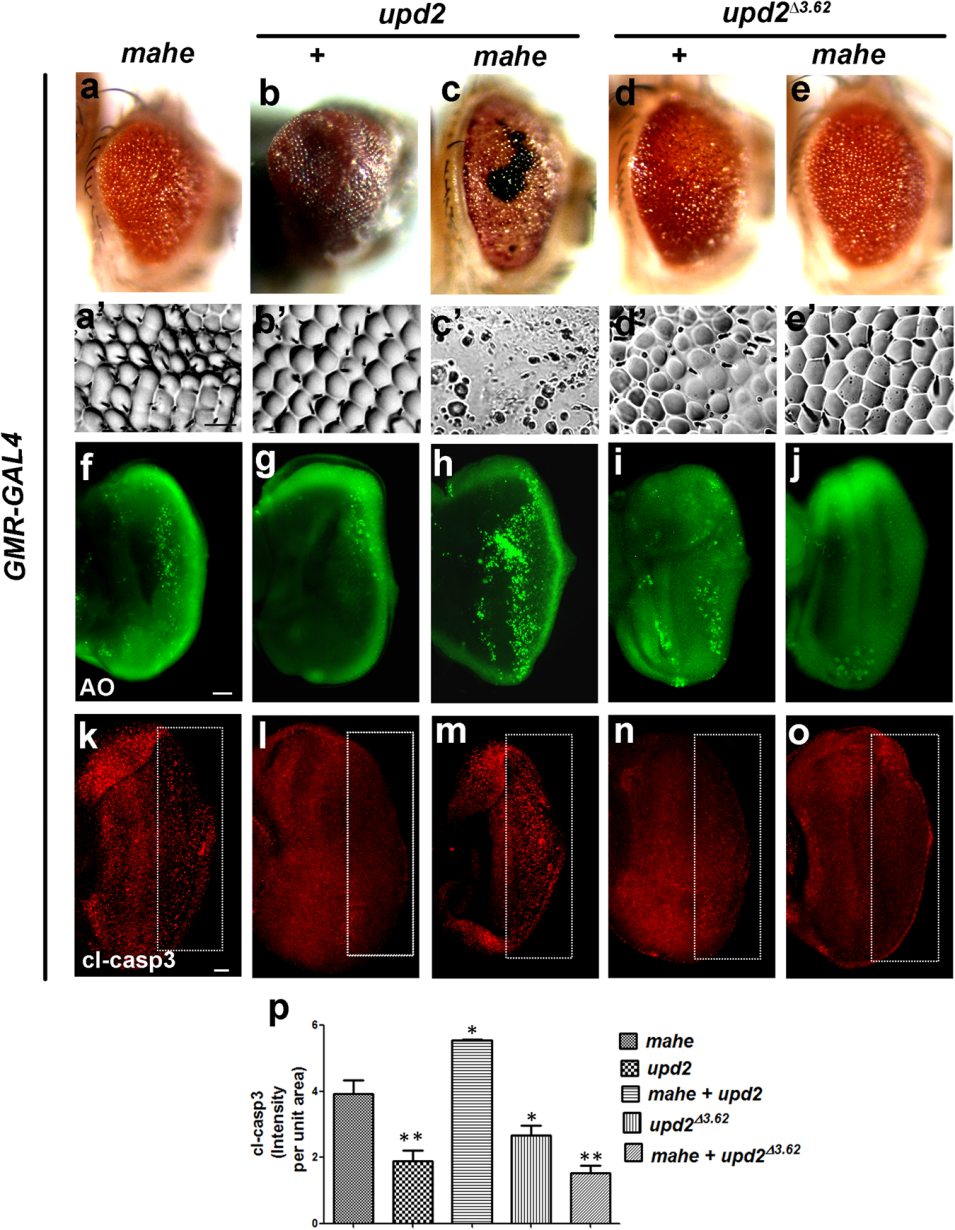
Ectopic expression of *upd2* enhances, while loss of *upd2* suppresses *mahe* induced apoptosis in developing eye. **a**–**e** Images of *Drosophila* adult eye. **a′–e′** Eye imprints of adult eye. *GMR-GAL4* was used to drive the expression of *UAS-mahe* (**a**, **c**, **e**) or *UAS-upd2* (**b**, **c**)**. a**, **a′** *GMR-GAL4* > *mahe* promotes eye roughening. **b, b′** *GMR-GAL4* driven *upd2* resulted in severely overgrown eyes with regular patterned ommatidia. **c**, **c′** Coexpression of *upd2* and *mahe* leads to pupal lethality whereas few escapers showed drastic eye roughness, with loss of pigmentation, black necrotic patches and loss of ommatidia in comparison to ectopic expression of *mahe* alone. **d**, **d′** *upd2*^*∆3-62*^ a loss-of*-*function allele shows slightly disorganized and fused ommatidia along the dorsal side of adult eye. **e**, **e′** *upd2*^*∆3-62*^ in combination with *GMR-mahe* suppressed the disorganized adult eye phenotype. **f**–**j** and **k**–**o** Acridine orange and caspase staining (compare area marked within rectangle) in eye-antennal disc of third instar larvae. **f**, **k** Acridine orange and caspase staining marked dying cells on ectopic expression of *mahe*. **g**, **l** Fewer acridine orange and caspase positive cells were observed upon *upd2* overexpression. **h**, **m** Coexpression of *upd2* and *mahe* enhanced cell death associated with *mahe*, which is reflected by increase in acridine orange and caspase positive cells when compared to *mahe* alone. **i**, **n** *upd2*^*∆3-62*^ showed fewer acridine and caspase positive cells. **j**, **o** *upd2*^*∆3-62*^ rescued the *mahe* induced cell death, which is depicted by reduced number of acridine orange and caspase positive cells. **p** Graph represents cl-caspase intensity per unit area which shows *upd2* overexpression promotes *mahe* induced cell death, and was significantly reduced in combination with *upd2* mutant. One*-*way ANOVA. Scale bar in 50 µm (**a′**–**e′**, **f**–**j**, **k**–**o**). Genotypes (**a**) *w/+; +; GMR-GAL4,UAS-HA-mahe/+* (**b**) *w/+; UAS-upd2:GFP/*+*;GMR-GAL4/*+ (**c**) *w/+;UAS-upd2:GFP/*+*;GMR-GAL4,UAS-HA-mahe/+* (**d**) *upd2*^*∆3.62*^*/+; +;+* (**e**) *upd2*^*∆3.62*^*/+; +; GMR-GAL4, UAS-HA-mahe*. One-way ANOVA * *P* < 0.05, ** *P* < 0.01, *** *P* < 0.001.

Since, major components of the JAK/STAT pathway were upregulated as depicted in the RNA-Sequencing data, we next sought to identify whether the components of JAK/STAT pathway themselves alter the *mahe* induced apoptotic phenotype. Coexpression of both *mahe* and *upd2* exhibited severe disorganization of ommatidia with black necrotic patches that were observed in a few of the escapers, while most of the pupae failed to eclose and were dead. (Fig. 3c–c′). This was in contrast to the rough and enlarged eye phenotype exhibited by overexpression of *mahe* and *upd2* alone (Fig. 3a, a′ and b, b′). Both, acridine orange and cleaved caspase immunostaining showed dramatic enhancement in apoptosis when *upd2* and *mahe* were coexpressed in the developing eye-antennal discs (Fig. 3h, m to f, k and g, l). On similar lines, heterozygous *upd2* mutant (Fig. 3d, d′, i, n) in combination with *mahe* overexpression completely rescued the apoptotic phenotype (Fig. 3e, e′, j, o to a, a′, f, k). Quantitative analysis of cleaved caspase staining showed enhancement of apoptotic cells, when both *upd2* and *mahe* were expressed together, whereas in contrary *mahe* induced phenotype was suppressed in heterozygous combination with *upd2* loss of function allele (Fig. 3p).

We also observed that *upd1* ligand that activates JAK/STAT signaling^17^ also enhanced *mahe* induced apoptosis (Fig. S1). Our observation was supported by an independent transgenic RNA interference (RNAi) screen performed for *mahe* modifiers (unpublished Pandey and Mutsuddi). Downregulation of Chaperonin containing TCP1 subunit 7 (*CCT7*) gene, which is known to be a negative regulator of *upd1*^18^ led to enhancement of apoptosis induced by *mahe* (Fig. S2) suggesting, a positive role of *mahe* induced JAK/STAT signaling in promoting cell death. Our genetic interaction studies are in agreement with our transcriptome data. Our results clearly display that ectopic expression of *upd2* dramatically enhances the apoptotic phenotype of *mahe*, while moderate enhancement of apoptosis was seen in combination of *upd1* and *mahe*. Reduced levels of both *upd1* and *upd2* led to massive suppression of the *mahe* induced phenotype. This suggested a vital role of JAK/STAT signaling in *mahe* induced cell death.

### *hopscotch* transcripts coimmunoprecipitates with Mahe and this interaction leads to its stabilization

In *Drosophila, upd* acts through the Dome/Hop/STAT92 signaling pathway for proper photoreceptor development^14,19^. We were further interested to check how *mahe* may be involved in altering JAK/STAT signaling. To determine the mechanism that connects *mahe* with JAK/STAT pathway-mediated apoptosis, we hypothesized that Mahe which encodes an RNA binding protein, might physically interact with the transcripts of any/some of the major components of JAK/STAT pathway via its RNA binding domain thereby ectopically activating and enhancing JAK-STAT signaling output.

**Fig. 4:**
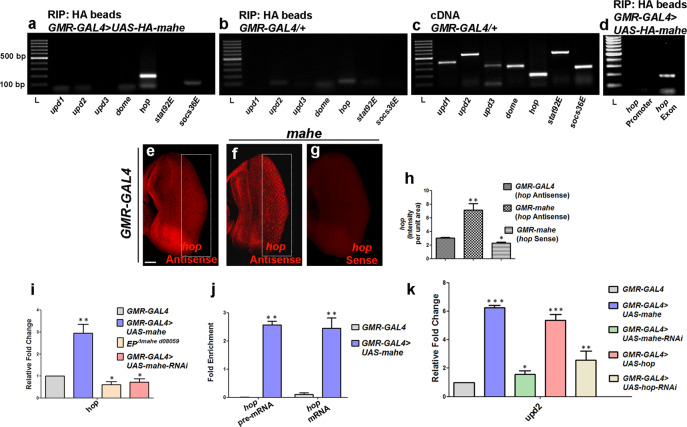
Mahe directly binds to *hop* transcripts and increases/stabilizes its level leading to upregulation of *upd2* ligand. **a**, **b**, **d** RNA immunoprecipitation was done using Anti-HA beads to immunoprecipitate HA-tagged-Mahe along with associated RNA-protein complex. **a** Protein lysates from *GMR-GAL4* > *UAS-HA-mahe* was used for immunoprecipitation followed by RT-PCR with primers specific for components of JAK/STAT pathway. Out of the different components *upd1*, *upd2*, *upd3*, *dome*, *hop*,*stat92E*, and *socs36E* of JAK/STAT signaling, only *hop* was amplified indicating that Mahe binds with *hop* transcripts. **b** No amplification was observed in negative control in which *GMR-GAL4/*+ lysate was used for immunoprecipitation. **c** cDNA samples without RNA immunoprecipitation were used for positive control. **d** No amplification was observed with primer specific for *hop* promoter, while *hop* exon specific primers showed positive amplification. **e**–**g** RNA-FISH was done to check the level of *hop* transcripts in eye antennal discs. **e** FISH with *hop-specific* antisense riboprobe in wild-type eye antennal disc showed a very little signal. **f** Ectopic *mahe* expression revealed enhanced levels of *hop* transcript posterior to the morphogenetic furrow when compared to that of the control (area in rectangle). **g** *hop* sense riboprobe was used as negative control and no signal was seen in *mahe* overexpressed eye-antennal discs. **h** Graph represents *hop* intensity per unit area that clearly shows *mahe* overexpression leads to increase in *hop* transcript levels when compared to that of wild-type control. One-way ANOVA. **i** Quantitative real-time PCR shows increase in *hop* transcripts level upon *mahe* overexpression when compared to transcripts from *GMR-GAL4* control tissue. One-way ANOVA. **j** Enrichment of both *hop* pre-mRNA and mRNA was observed by real-time PCR in RIP precipitate. One-way ANOVA. **k** Overexpression of *hop* alone leads to increase in *upd2* transcript levels, similar to that of ectopic *mahe*. One-way ANOVA. Scale bar in 50 μm (**e**–**g**). Genotypes (**b**, **c**, **e**, **h**, **i**, **j**, **k**) *w/+; +; GMR-GAL4/*+ (**a**, **d**, **f**, **g**, **h**, **i**, **j**, **k**) *w/+; +; GMR-GAL4,UAS-HA-mahe/UAS-HA-mahe* (**i**, **k**) *w/+; UAS-mahe-RNAi/+; GMR-GAL4/*+ (**k**) *w/+; UAS****-****hop/+; GMR-GAL4/*+ (**k**) *w/+; UAS****-****hop-RNAi/+; GMR-GAL4/*+. One-way ANOVA * *P* < 0.05, ** *P* < 0.01, *** *P* < 0.001.

To test this hypothesis, immunoprecipitation of RNA-protein complex was carried out from fly head extracts using anti-HA beads to pull down HA-tagged Mahe, while RNA-protein extracted from *GMR-GAL4* alone served as negative control. This was followed by RT-PCR using gene-specific primers for the members of JAK/STAT pathway, *upd1*, *upd2*, *upd3*, *dome*, *hop*, *stat92E*, and *socs36E*. Interestingly, anti-HA pulled down HA-Mahe was positive only for the presence of *hop* transcripts that encode the tyrosine kinase involved in JAK/STAT signaling (Fig. 4a–c). Further, a complete absence of amplification with *hop* promoter-specific primers and positive amplification with *hop* exon-intron boundary-specific primers ensured the specificity of *hop* RNA and Mahe interaction. (Fig. 4d). This ruled out the interaction of Mahe RNA helicase with that of the promoter region or the DNA of *hop*.

Additionally, fluorescent RNA in situ hybridization (FISH) with *hop-specific* antisense RNA probe was done in *mahe* overexpressed eye-antennal discs to visualize, whether the interaction of *hop* transcripts with Mahe led to any change in the turnover rate of *hop* transcripts. Increase in *hop* transcript levels was seen in *mahe* overexpressed discs in comparison to the wild-type control discs, whereas no signal was observed with the *hop* sense probe (Fig. 4e–h). For unchanged control *hsrω* probe was used, and no change in transcript levels was observed in eye antennal discs overexpressing *mahe* when compared to *GMR-GAL4* control (Fig. S3). This observation clearly signifies that recruitment of *hop* transcripts in the Mahe protein complex leads to stabilization and increase in *hop* RNA levels. RT-PCR analysis was also in agreement with our FISH data (Fig. 4i). The next question was whether *mahe* enhanced the levels of processed *hop* transcripts or pre-mRNA. RT-PCR using *hop* pre-mRNA and mature RNA-specific primers identified the presence of both types of RNAs in the ribonucleoprotein complex (Fig. 4j). It is likely that the interaction of *hop* transcripts with Mahe RNA helicase reduces the turnover rate of transcripts and stabilizes it, thus resulting in elevated levels of *hop* RNA. We further hypothesized that ectopically elevated *hop* transcripts might stimulate upregulation of *upd2* ligand which in turn promotes a positive feedback signal for ensuring prolonged JAK/STAT activation. Our proof of principle was strengthened by the elevated levels of *upd2* RNA in tissues where *hop* was overexpressed (Fig. 4k).

**Fig. 5:**
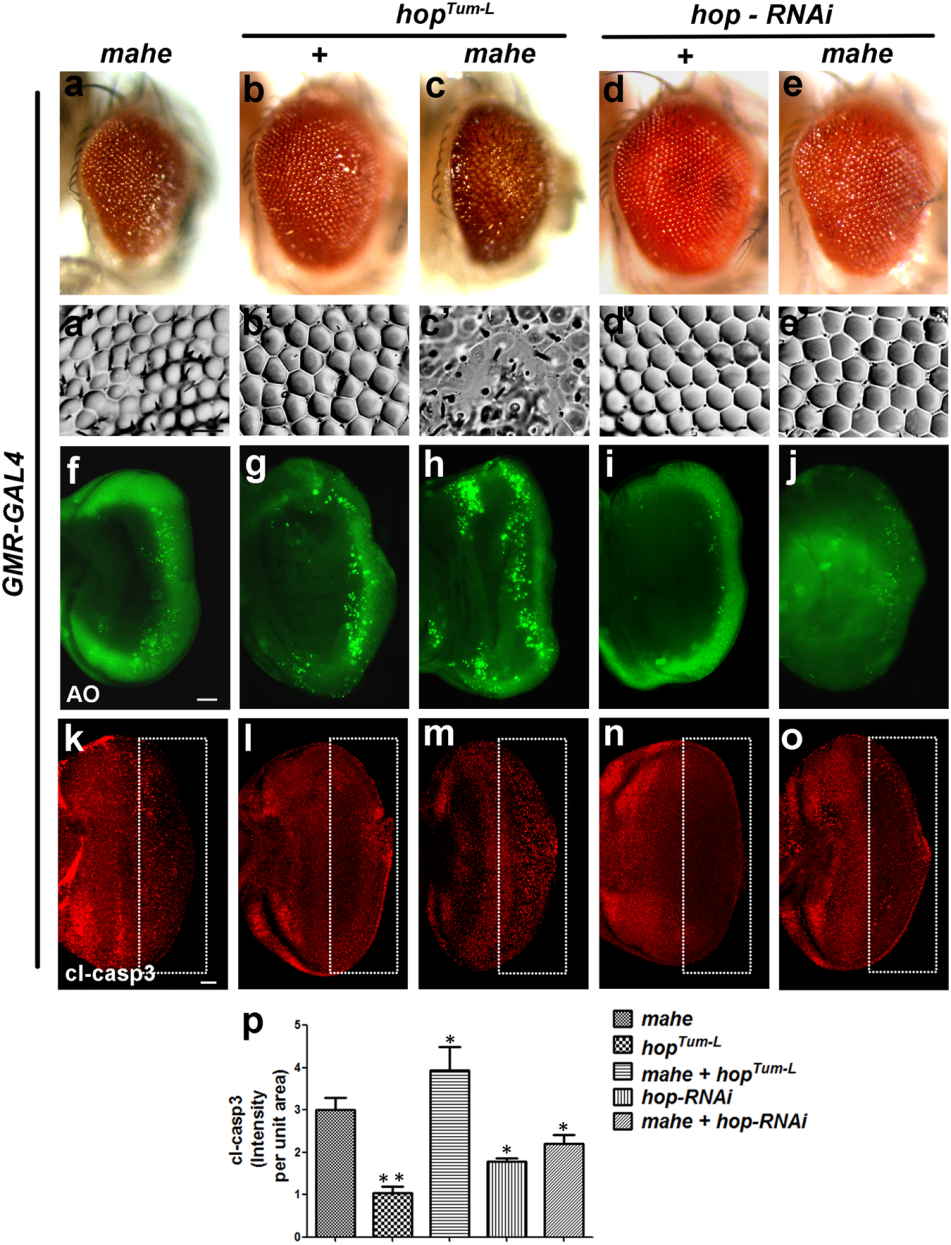
*hopscotch* enhances *mahe* induced cell death. (**a**–**e**) Images of *Drosophila* adult eye. **a′**–**e′** Adult eye imprints. **a**, **c**, **e** *GMR-GAL4* was used to drive *UAS-mahe* or (**b**, **c**) *UAS-hop*^*(Tum-l)*^ (**d**, **e**)*UAS-hop-RNAi*. **a**, **a′** Ectopic *mahe* expression promotes eye roughening. **b**, **b′** Overexpression of constitutive active form of *hop* results in slight ommatidial disorganization. **c**, **c′** Coexpression of *hop* and *mahe* enhanced the rough eye phenotype compared to *mahe* alone. **d**, **d′** Reduction of *hop* levels by RNAi mediated knockdown leads to no observable change in eye roughening phenotype. **e**, **e′** RNAi mediated depletion of *hop* rescued the *mahe* induced eye roughness. **f**–**j**, **k**–**o** Acridine orange and caspase staining in eye antennal disc. **f**, **k** Acridine and caspase positive cells show cell death induced by *mahe*. **g**, **l** *hop*^*Tum-l*^ overexpression showed few dying cells. **h**, **m** Coexpession of *hop*^*Tum-l*^ and *mahe* enhanced cell death in comparison to *mahe* alone. **i**, **n** No dying cells were observed on downregulating the level of *hop.* **j**, **o** Downregulation of *hop* rescued the *mahe* induced cell death as depicted by acridine orange and cl-caspase staining. **p** Graph represents cl-caspase intensity per unit area which shows activated *hop*^*Tum-l*^ promotes *mahe* induced cell death, which was significantly reduced in combination with *hop-RNAi*. One-way ANOVA. Scale bar in 50 µm (**f**–**j**, **k**–**o)**. Genotypes (**a**) *w; +; GMR-GAL4,UAS-HA-mahe/+* (**b**) *w; UAS-hop*^*Tum-l*^*/+;GMR-GAL4/*+ (**c**) *w; UAS-hop*^*Tum-l*^*/+;GMR-GAL4,UAS-HA-mahe/+* (**d**) *w; UAS-hop-RNAi/+;GMR-GAL4/*+ (**e**) *w; UAS-hop - RNAi/+; GMR-GAL4,UAS-HA-mahe/+*. One-way ANOVA * *P* < 0.05, ** *P* < 0.01, *** *P* < 0.001.

To further establish the link between active *hop* and *mahe*, we carried out genetic interaction with hyperactive JAK Kinase *UAS-hop*^*(Tum-l)*^ which carries a dominant mutation in *hop* that activates Stat92E and with *UAS-hop-RNAi*. Coexpression of *GMR-GAL4* driven *mahe* and *UAS-hop*^*(Tum-l)*^ resulted in an enhanced rough eye phenotype as compared to that of expressing both the components by themselves (Fig. 5c, c′ to a, a′ and b, b′). Further, extensive cell death was detected posterior to the morphogenetic furrow that was depicted by acridine orange and anti-caspase antibody immunostaining in eye-antennal discs with overexpressed *hop* and *mahe* (Fig. 5h, m to f, k and g, l, p). In line with above genetic interaction, conversely downregulation of *hop* led to suppression of *mahe* induced apoptotic phenotype (Fig. 5e, e′, j, o to a, a′, f, k and d, d′, i, n). Thus, our molecular and genetic interaction studies suggest that interaction of Mahe with *hop* RNA stabilizes the *hop* transcripts, leading to the activation of JAK/STAT pathway, which in turn induces apoptosis.

### Increase in both cytoplasmic and nuclear *Stat92E* levels indicates activation of JAK/STAT pathway that ultimately leads to *hid* mediated apoptosis

**Fig. 6:**
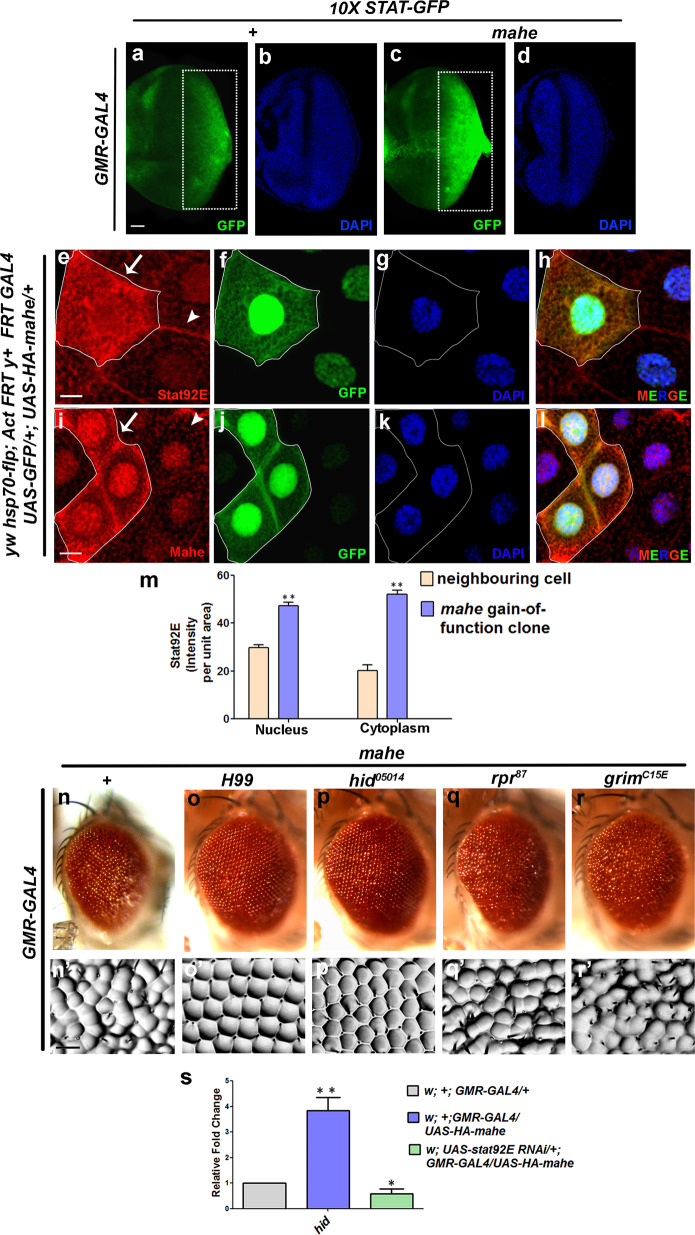
JAK/STAT pathway activation by ectopic *mahe* induces *hid* dependent apoptosis. **a**–**d** Activation of JAK/STAT pathway was detected by *10X STAT92E-GFP* reporter in eye-antennal discs. **a**, **c** Significant increase in GFP level was observed in *GMR-GAL4* driven *mahe* eye antennal disc when compared to the reporter line alone, indicating activation of JAK/STAT signaling (compare area in rectangle). **b**, **d** DAPI was used to mark the nucleus. **e**–**l** Gain of function clone of *mahe* displays enhanced Stat92E activity. Gain of function clones of *mahe* using *UAS-HA-mahe* flies were generated with FLP/FRT system in salivary gland. **f**, **j** GFP positive and non-GFP cells mark *mahe* gain-of-function clones (marked with arrow) and wild-type cells (marked with arrowhead), respectively. **e** Level of Stat92E the transcription factor for JAK/STAT signaling was enhanced in *mahe* gain-of function somatic clones when compared to wild-type neighboring cells. **i** Clonal area marked with GFP positive cells, shows elevated level of Mahe in the gain-of-function clones. **g**, **k** DAPI was used to mark the nucleus. **h**, **l** Fourth column represents merged image with DAPI staining. **m** Graph represents Stat92E intensity per unit area which shows marked enhancement in the level of Stat92E in nucleus and cytoplasm, in *mahe* overexpressing clones when compared to neighboring cells. One-way ANOVA. **n**, **n′** Ectopic expression of *mahe* results in rough eye phenotype. **o**, **o′** and **p**, **p′** *mahe* induced rough eye phenotype was rescued by *H99* and *hid* mutant, but not with *rpr* and *grim* mutants (**q**, **q′** and **r**, **r′**). **s** Real time PCR showed a threefold increase in *hid* transcript levels upon ectopic expression of *mahe*, while downregulation of JAK/STAT pathway by *stat92E-RNAi* rescued the *hid* levels. One-way ANOVA. Scale bar in 50 µm (**a**–**d**, **e**–**l**). Genotypes (**a**, **b**) *w/+; 10X STAT-GFP/*+*, GMR-GAL4/*+ (**c**, **d**) *w/+; 10X STAT-GFP/*+*; GMR-GAL4, UAS-HA-mahe/+* (**n**) *w/+; +; GMR-GAL4,UAS-HA-mahe/+* (**o**) *w/+; +; GMR-GAL4,UAS-HA-mahe/H99* (**p**) *w/+; +; GMR-GAL4,UAS-HA-mahe/hid*^*05014*^ (**q**) *w/+; +; GMR-GAL4,UAS-HA-mahe/rpr*^*87*^ (**r**) *w/+; +; GMR-GAL4,UAS-HA-mahe/grim*^*C15E*^. One-way ANOVA * *P* < 0.05, ** *P* < 0.01, *** *P* < 0.001.

To investigate the role of *mahe* in activating JAK/STAT signaling we checked the status of the most downstream component Stat92E, a transcription factor required for JAK/STAT signaling. To test this, we utilized *10X-Stat92E-GFP* line containing five tandem repeats of *socs36E* binding sites, a transcriptional reporter for JAK/STAT signaling^20^. As predicted, we observed a surge in *Stat92E-GFP* reporter activity upon ectopic expression of *mahe*, when compared to that of the basal Stat92E reporter gene expression (Fig. 6a–d). This supported our transcriptome data where we saw elevated *socs36E* transcripts, which is a downstream target of Stat92E (Fig. 1e, f). The above results further strengthened our findings that JAK/STAT pathway is activated and upregulated by *mahe*.

We argued that an elevated expression of Stat92E protein can be correlated with increase in activation of JAK/STAT pathway. We assessed the expression of Stat92E protein in gain-of-function *mahe* clones to check whether the levels of STAT92E protein were altered in the somatic clones. The stock *yw hsp70-flp; Act FRT y*^*+*^ *FRT-GAL4 UAS-GFP/*+*;* +, was used to generate clonal cells in the salivary gland, in which *mahe* levels were enhanced with *UAS-HA-mahe* (Fig. 6e–l). The somatic clones with enhanced Mahe expression were marked with GFP (Fig. 6i–l). A substantial increase in Stat92E expression in the nucleus and cytoplasm was observed in *UAS-HA-mahe* clones, whereas in the non-clonal cells, the fluorescent signal was comparatively lower (Fig. 6e–h, m). We also made an effort to generate *mahe* loss-of-function mutant clones using FLP-FRT system; however, mutant clones of *mahe* did not survive and hence we were unable to carry out *mahe* loss of function analysis. However, the accumulation of Stat92E protein in *mahe* overexpression clones is a strong proof that *mahe* leads to activation of JAK/STAT pathway.

It has been previously reported that *upd* via JAK/STAT signaling promotes apoptosis through *hid* in supernumerary polar cells during *Drosophila* oogenesis^21^. Suppression of *mahe* rough eye phenotype by *DIAP1* (Fig. 2), led us to examine the effect of proapoptotic genes in *mahe* mediated apoptosis. *H99* mutant in which all the three genes *hid, rpr* and *grim* are deleted, in a heterozygous combination led to suppression of ectopic *mahe* induced phenotype. We then carried out genetic interaction analysis with mutants of proapoptotic genes. It was observed that mutations in *hid*, but not in *rpr* and *grim* suppressed *mahe* induced rough eye phenotype (Fig. 6n–r and n’- r’). In addition, a 3 fold increase in *hid* transcript levels were observed with *mahe* overexpression, supporting the involvement of *hid* in *mahe* induced cell death (Fig. 6s).

Next, we quantified *hid* transcript levels after blocking JAK/STAT activity. Lowering levels of *Stat92E* via RNAi in *mahe* overexpression background, led to a 2.5 fold decrease in *hid* transcripts in comparison to transcripts from *mahe* overexpressed tissue (Fig. 6s). This clearly indicated that *mahe* positively regulates JAK/STAT pathway, which ultimately modulates the levels of *hid* transcripts and lowering of the downstream effector *Stat92E* alone was sufficient to rescue the *hid* mediated apoptosis.

**Fig. 7:**
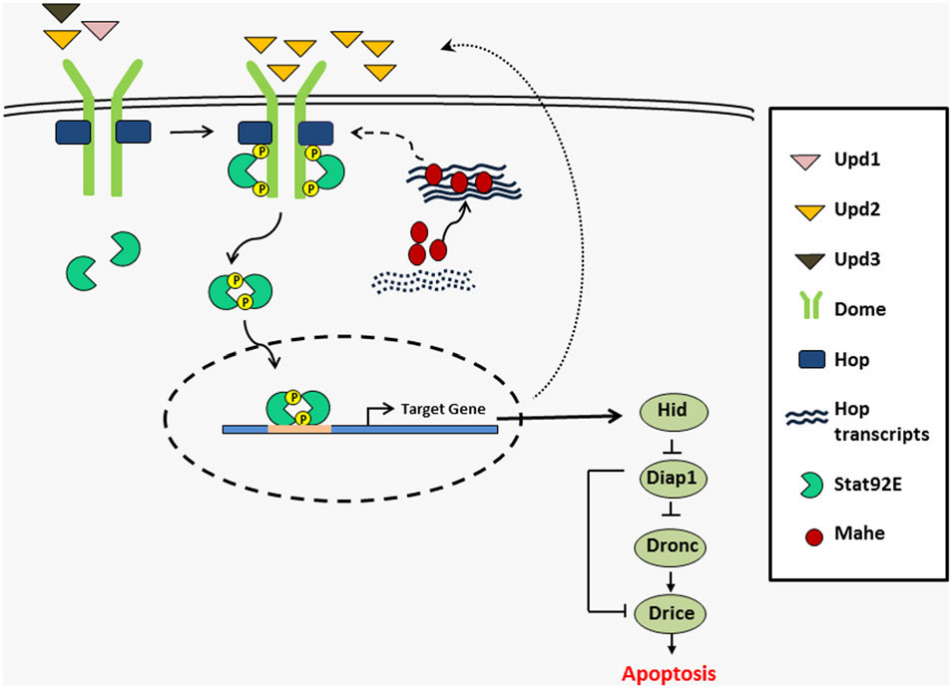
A hypothetical model depicting recruitment and stabilization of *hop* kinase transcripts by RNA helicase Mahe which leads to activation of JAK/STAT signaling and primes *hid* mediated apoptosis. Our model depicts that Mahe encoding an RNA helicase binds and regulates *hop* transcript stability, and this results in the increase in levels of Hop kinase. This upregulation of Hop tyrosine kinase leads to enhanced phosphorylation and activation of transcription factor Stat92E which enters into the nucleus, resulting in downstream target gene activation. An auto feedback loop is reflected by increase in the levels of Upd2, and thus the JAK/STAT signaling remains in an active state. This activated JAK/STAT signaling then induces *hid* mediated apoptosis.

Based on our findings we put forward a hypothetical model (Fig. 7), where we propose that Mahe regulates JAK/STAT signaling by directly interacting with *hop* tyrosine kinase transcripts leading to its stabilization and this cascade the activation of downstream effector molecules in this pathway. This in turn leads to activation of proapoptotic gene *hid*, along with a surge in apoptosis.

## Discussion

A number of reports have shown that Decapentaplegic (Dpp), Hedgehog (Hh), Notch, JAK/STAT and Wingless (Wg) signaling pathways are major regulators of development and differentiation of photoreceptor neurons in *Drosophila*^22–28^. Dysregulation of JAK/STAT pathway has been implicated in a wide variety of neurological disorders like the glial tauopathy and Parkinson’s Disease^29–31^. We have recently reported that mutation in *DDX59*, the human homolog of *maheshvara* (*mahe*), is associated with neurological abnormalities and is crucial for development of the nervous system^11^.

In this report we have elucidated a novel link between *mahe* and JAK/STAT signaling, suggesting the significance of *mahe* in regulation of JAK/STAT signaling in the photoreceptor neurons of *Drosophila*. Our transcriptome analysis has identified differentially regulated genes that are predominantly related to neuronal function, phototransduction, stress response and regulation of cell signaling. Interestingly, by quantitative-PCR analysis, we found that all the ligands and downstream effector of JAK/STAT pathway were significantly lowered in *mahe* loss-of-function mutant and in *mahe-RNAi*. Similarly, upregulation of the pathway components suggested ectopic activation of the JAK/STAT signaling by overexpressed *mahe*, which was in agreement with the gene ontology analysis of transcriptome data.

JAK/STAT signaling is an important pathway required for *Drosophila* eye development and for proper axon targeting in a cell autonomous manner during photoreceptor formation^32^. Further, genetic studies have previously shown that overexpression of *upd and hop*^*Tumorous-lethal*^ which encodes constitutively active form of *hop* results in dramatically overgrown adult eyes. In addition, downregulation of JAK/STAT pathway by hypomorphic *upd* mutant and dominant negative form of *dome*^*∆cyt*^ leads *to* small or ablated eyes^19,22,33–35^. However, in contrast in *lobe* mutants, ectopic *upd* expression is known to activate JAK/STAT pathway and induce apoptosis, which leads to small eye phenotype^36^. Taken together these findings suggest that JAK/STAT pathway can induce both context-dependent proliferation as well as apoptosis and is needed for proper eye development in *Drosophila*. Although both *upd2* and *upd3* are ligands for JAK/STAT pathway but their role in *Drosophila* photoreceptor development, specifically cell death has not been investigated. Here, we observed that coexpression of both *mahe* and *upd2* along with other components of JAK/STAT pathway results in massive apoptosis. Our findings reveal that *mahe* activated JAK/STAT signaling can induce apoptosis in *Drosophila* photoreceptors thus showing context-dependent role of JAK/STAT pathway during development. Conversely, loss of JAK/STAT signaling components leads to rescue in the *mahe*-induced apoptosis.

RBPs are known to modulate post-transcriptional regulation, turnover, localization, and translational control of mRNA. Reports have shown that RBPs can act in both positive or negative manner to stabilize mRNA^37,38^. *TTP* and *KSRP* destabilizes several mRNA like *c-fos*, *TNFα* and *COX-2*, however *HuR* acts positively to maintain their stability^39–42^. A recent report revealed that RNA binding protein Musashi 2 (MSI2) regulates IL-6 signal transducer (IL6ST) and promotes its degradation by binding to 3′UTR of mRNA. IL6ST cytokine receptor in turn after forming complex with kinases affects the phosphorylation of STAT3 and other mitogen-activated protein kinase ERK^43^. Similarly, in order to understand the mechanism of ectopic JAK/STAT activation, we argued whether Mahe a DEAD box RNA helicase might interact directly with the transcripts of the components of JAK/STAT transduction cascade to regulate the pathway. Interestingly, *hop* transcripts were found to be associated with Mahe-containing RNA–protein complex. We propose a model in which this interaction may lead to decrease in the turnover rate of transcripts, and thus leads to stabilization and elevation in *hop* transcripts. The elevated JAK is activated possibly via interaction with the receptor and ligand Upd2 in response to ectopic *mahe*. Mechanism that could account for activation of the pathway is probably via augmented phosphorylation and activation/stabilization of downstream transcription factor Stat92E, as seen by its enhanced cytoplasmic to nuclear localization. Next, we hypothesized that this interaction can result in enhanced *upd2* RNA levels, which is further supported by a significant increase in *upd2* transcript levels upon expression of *hop* alone. Our prediction that *mahe* activates downstream targets of JAK/STAT signaling was supported by our reporter gene analysis using *10X Stat92E GFP* line. Further, a massive increase in both cytoplasmic and nuclear Stat92E transcription factor was seen in *mahe* gain-of-function somatic clone. Activation of the JAK/STAT pathway was further reflected by the increase in transcription of downstream target of *socs36E*. The above finding was supported by a report where oral infection initiates immune response in the gut of *Drosophila* followed by upregulation of JAK/STAT signaling, and the pathway activity was assessed by transcript levels of *socs36E*^44^. Based on these findings we propose that Mahe and *hop* interaction upregulates the JAK/STAT pathway via an auto feedback loop.

In summary, we report that *mahe* induces JAK/STAT signaling mediated apoptosis during *Drosophila* photoreceptor development. We put forth a novel mechanism in which *hop* is post-transcriptionally regulated by a DEAD box RNA helicase Maheshvara, which ultimately results in activation of JAK/STAT signaling mediated apoptosis. As deregulated JAK/STAT signaling and apoptosis have been linked to many human diseases, the identification of a novel regulator of this pathway will lead to a better understanding of development and disease. Identification of Maheshvara as a novel modulator of JAK/STAT pathway has opened up a novel platform for understanding the role of RNA helicase associated cell death which may also hold good for human disorders as well.

## Material and methods

### *Drosophila* genetics

All the fly stocks were maintained on standard cornmeal/yeast/molasses/agar medium at 25 °C. *w*^*1118*^ was used as the wild-type control. *UAS-HA-mahe*^10^ and *EP*^*∆mahe d08059*^^11^ were generated in our lab. *UAS-upd2-GFP* and *upd2*^*∆3-62*^were kind gift from Prof. James Castelli-Gair Hombría. *UAS-mahe-RNAi* (v109465), *UAS-hop-RNAi, UAS-stat92E-RNAi, UAS-upd1-RNAi, UAS-CCT7-RNAi* were obtained from VDRC*.GMR-GAL4*, *UAS-DIAP*, *UAS-P35*, *UAS-upd1*, *UAS-hop*, *10X STAT-GFP, H99, hid*^*05014*^*, rpr*^*87*^*, grim*^*C15E*^, stocks were obtained from Bloomington *Drosophila* Stock Center. All the crosses were performed at 25 °C, unless mentioned otherwise. To generate *mahe* gain-of-function clones females of *hsp70-flp; Act FRT y*^*+*^ *FRT-GAL4 UAS-GFP/*+*;* +*/*+ were crossed to *UAS-HA-mahe* males. Heat shock was given at 37 °C for 10 min at 24 h AEL and the third instar larvae were analyzed for GFP marked clones.

### Eye imprints

Eye imprints using nail polish were prepared for analysis of ommatidial defects and were examined under differential interference contrast (DIC) optics in a Nikon Eclipse 80i microscope.

### Acridine orange staining

To observe the extent of apoptosis we used the vital dye acridine orange (AO). Eye-antennal discs from larvae of desired genotypes were dissected in phosphate buffer saline (PBS) (130 mM NaCl, 7 mM Na_2_HPO_4_, 3 mM KH_2_PO_4_, pH 7.4) and stained with 1 μg/μl of Acridine orange (AO) in PBS for 3 min. Followed by two washes with PBS and were finally mounted in PBS. These were than immediately viewed under Nikon Eclipse 80i microscope.

### Immunostaining

Immunostaining was performed in various tissues dissected from third instar larvae. Larvae were dissected in PBS (pH 7.4) and immunostaining was done as described previously^10^. To mark the nuclei, staining with 4′, 6- diamidino-2-phenylindole dihydrochloride (DAPI) (1 µg/ml) was done. Tissues were mounted in DABCO. All slides were observed under LSM 780 laser scanning confocal microscope Zeiss (Carl Zeiss), Thornwood, NY. The images were further processed with Adobe Photoshop 7. The following primary antibodies were used in this study: rabbit anti-Mahe (1:300) generated in our laboratory, rabbit anti-cleaved Caspase 3 (1:100) (Cell Signalling Technology), rabbit anti-Stat92E (1:200) a kind gift from Erika Bach. Secondary antibody used was Alexa Fluor 555 conjugated goat anti-rabbit IgG (1:200). Intensity profile graphs were made by using Image J and Graph Pad Prism 5 software.

### RNA extraction and Real-time PCR

Total RNA was extracted using Trizol reagent (Invitrogen), from adult fly heads of desired genotypes. To remove genomic DNA contamination the extracted RNA was treated with RNase free DNaseI for 30 min at 37 °C. Reverse transcription was performed with a cDNA synthesis kit (Applied Biosystems, Foster City, CA), using aliquots of total RNA extracted. Real-time quantitative PCR was performed to check the expression of desired genes of interest. Real-time PCR reactions were performed using the ABI 7500 sequence detection system (Applied Biosystems) with SYBR Green PCR Master Mix (Thermo Scientific). The relative quantity of amplified cDNA/DNA corresponding to the gene was determined using the ΔΔCt method and normalized for expression of *rps17* in each sample^45^. The graph was prepared using Graph Pad Prism 5 software.

Primers used for the study are as follows:

*mahe* forward primer: 5′-TTCGTGCGTTGGCCCTTGTTATTG-3′

*mahe* reverse primer: 5′-GCTGGGCATCGAACGAGCAAG-3′

*upd1* forward primer: 5′- ATTGCCCTAAAGCGCTGGTACCG-3′

*upd1* reverse primer: 5′- GTAGTAGTGGTGCTTCACAAAGC-3′

*upd2* forward primer: 5′- GTGAAGCTAAAGACTTG-3′

*upd2* reverse primer: 5′- TCAAGACTCATTGGATCCGCCAT-3′

*upd3* forward primer: 5′-TGCCCCGTCTGAATCTCACT-3′

*upd3* reverse primer: 5′-GTGAAGGCGCCCACGTAA-3′

*hop* forward primer: 5′- GGGTATCTACATCAGATTGTC-3′

*hop* reverse primer: 5′-GCATTCACGCACAATATAGC-3′

*hop* promoter forward primer: 5′-CAAGAATATAGACGCCATAGAGC-3′

*hop* promoter reverse primer: 5′-GTCATCGATTGTCCAATA ACCTG-3′

*socs36E* forward primer: 5′- GCTGCCAGTCAGCAATATGT-3′

*socs36E* reverse primer: 5′- GACTGCGGCAGCAACTGT-3′

*hid* forward primer: 5′-AGC GTCTGCAGGAGTTCAAT-3′

*hid* reverse primer: 5′-CTTCGCCTTTTGTCGTTCTC-3′

*rps17* forward primer: 5′-AAGCGCATCTGCGAGGAG-3′

*rps17* reverse primer: 5′-CCTCCTCCTGCAACTTGATG-3′.

### Transcriptome analysis

Total RNA was isolated from 200 adult fly heads using standard Trizol method (Sigma) from adult fly heads of *GMR-GAL4* driven *UAS-mahe* and *GMR-GAL4* alone which served as control for comparison. Libraries were made using standard protocol of TrueSeq RNA sample Prep kit v2. Libraries were then sequenced by the paired-end reads using Illumina HiSeq2500 platform and the resulting sequencing reads were aligned to the reference *Drosophila melanogaster* genome downloaded from Ensemble database (ftp://ftp.ensembl.org/pub/release-81/fasta/drosophila_melanogaster/dna/Drosophila_melanogaster.BDGP6.dna.toplevel.fa.gz). The alignment was performed by STAR program (version = STAR_2.4.1d). Further, the aligned reads were used for estimating expression of transcripts using cufflinks program (version: cufflinks-2.2.1). The expression values are reported in FPKM (Fragment per kilo per million) units for each of the genes and transcripts. Differential expression analysis was performed using cuffdiffv2.2.1. Gene ontology analysis was done using DAVID 6.8 (The Database for Annotation, Visualization and Integrated Discovery).

### Immunoprecipitation of RNA-protein complex

Protein lysates were prepared by homogenizing 200 adult heads from 1 day old flies of *GMR-GAL4* driven *UAS-mahe* and *GMR-GAL4* alone which served as control for comparison in lysis Buffer (100 mM KCl, 5 mM MgCl2, 10 mM HEPES, pH 7.0, 0.5% Nonidet P-40, 1 mM DTT, 100 U ml^−1^ RNase inhibitor (NEB), 2 mM vanadyl ribonucleoside complexes solution (Sigma- Aldrich, 25 μl ml^−1^ protease inhibitor cocktail (Roche). Supernatant was collected and centrifuged at 12,000*g* for 20 min at 4 °C. Equilibration of anti-HA agarose beads was done in lysis buffer. Protein lysates were precleared by incubating with 20 μl of anti-HA agarose beads for 2 h at 4 °C. For immunoprecipitation, 50 μl of anti-HA beads were used for every 250 μl of protein lysate and were incubated for 5 h at 4 °C to pull down the desired protein of interest. This was followed by brief centrifugation to collect the beads. These beads were washed with 0.5 ml of polysome lysis buffer thrice by centrifugation at 2000*g* for 5 min at 4 °C. Washed beads were resuspended in 100 μl of lysis buffer with 30 μg of proteinase k and 0.1% SDS followed by heating at 50 °C for 30 min in order to degrade the protein. Total RNA was extracted using Trizol reagent. Upper aqueous phase having RNA was recovered by centrifugation and to it 10 μl of yeast tRNA (1 mg ml^−1^), 12 μl of 3 M sodium acetate and 250 μl of ethanol were added per 100 μl of aqueous phase and kept at −20 °C overnight for precipitation. This was followed by centrifugation at 12,000*g* for 20 min at 4 °C, ethanol was removed and the pellet rinsed with 70% ethanol followed by air drying until all the liquid evaporated. The pellet was resuspended in nuclease free MQ water. cDNA was synthesized from total RNA using M-MuLV Reverse Transcriptase (New England Biolabs) as per the manufacturer’s instructions. RT-PCR was performed using primers for *upd1*, *upd2*, *upd3*, *dome*, *hop*, *stat92E*, and *socs36E* listed above.

### RNA: RNA in situ hybridization

RNA-RNA in situ hybridization was carried out in tissue from third instar larvae. Larvae were dissected and RNA:RNA in situ hybridization was carried out as described earlier^37^. Hybridization was done with 100 ng *hop* antisense riboprobe in hybridization buffer at 50 °C for 12–16 h. *hop* sense riboprobe was used as negative control and was hybridized at 50 °C for 12–16 h. *hsrω* antisense riboprobe served as positive control and was hybridized for 12–16 h at 50 °C. For fluorescent detection of the riboprobe, anti-DIG-Rhodamine conjugated antibody (1:200, Roche) was used. DAPI was used to mark the nucleus. Samples were mounted in DABCO and observed under LSM 780 laser scanning confocal microscope Zeiss (Carl Zeiss). All the images were further processed using Adobe Photoshop 7.0.

### Statistical analysis

In our study experiments were conducted in triplicate, analysis was done using PRISM 5 as guided (GraphPad, San Diego, CA) and results were given as the mean ± standard deviation (S.D.). The statistical analysis was performed using one-way ANOVA. The significant level was set as *p*-values below 0.05.

## Supplementary information

Supplementarry Information
