# Supplementary material for: Maheshvara regulates JAK/STAT signaling by interacting and stabilizing hopscotch transcripts which leads to apoptosis in Drosophila melanogaster
Source: Cell Death Dis. 2021 Apr 6;12(4):363. doi: 10.1038/s41419-021-03649-0 (PMC8024297; doi:10.1038/s41419-021-03649-0)
Supplement: Supplementary file 1 — Supplementarry Information [file 41419_2021_3649_MOESM1_ESM.docx]

**Supplementary Information**


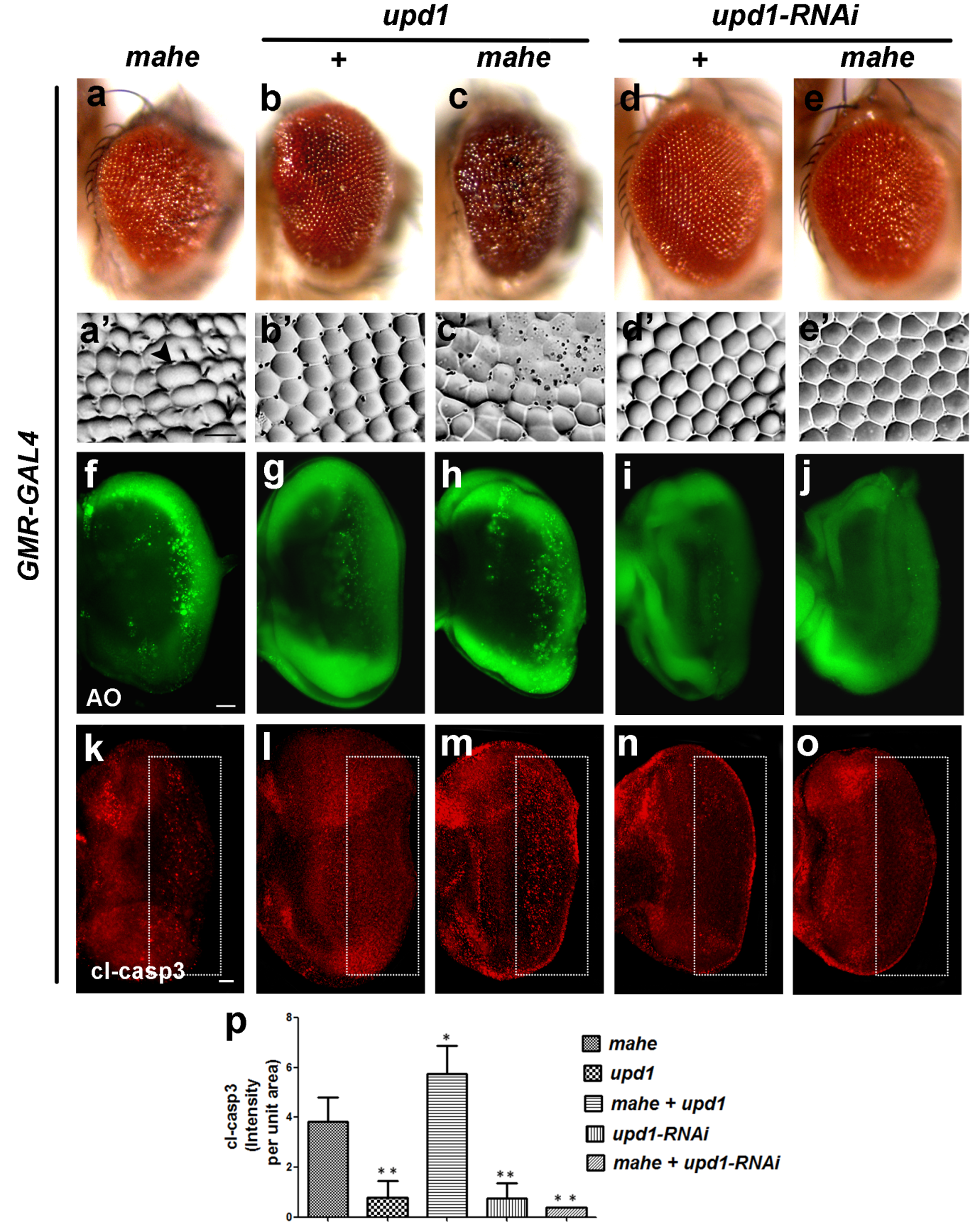


**Supplementary Figure S1: *upd1* genetically interacts with *mahe* and induces apoptosis.** *GMR-GAL4* was used to drive *UAS-mahe* **(a, c, e)** or *UAS-upd1* **(b, c)** or *UAS-upd1-RNAi* **(d, e). (a-e and a’-e’)** *UAS-upd1* enhanced while *upd1-RNAi* rescued the *mahe* induced eye roughness (**f-j, k-o and p)** Acridine orange and caspase staining showed *upd1* enhanced, while *upd1-RNAi* rescued apoptosis induced by *mahe* expression. Scale bar in 50µm **(f-j, k-o)**. Genotypes **(a)** *w; +; GMR-GAL4,UAS-HA-mahe/+* **(b)** *w; UAS-upd1/+;GMR-GAL4/+* **(c)** *w; UAS-upd1/+; GMR-GAL4, UAS-HA-mahe/+* **(d)** *w; UAS-upd1-RNAi/+;GMR-GAL4/+* **(e)** *w; UAS-upd1-RNAi/+;GMR-GAL4,UAS-HA-mahe/+.*


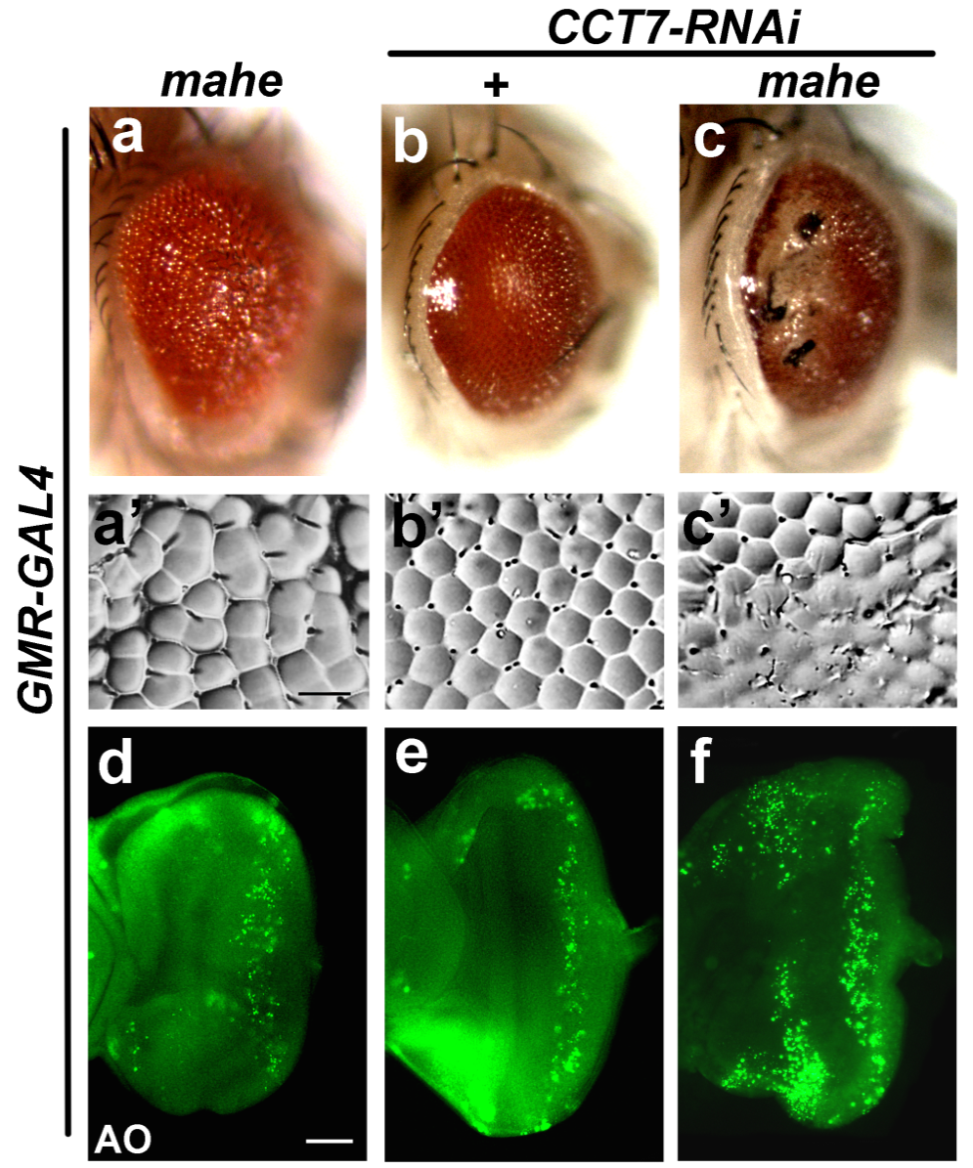


**Supplementary Figure S2: Downregulation of *CCT7* enhances *mahe* induced cell death.** *GMR-GAL4* was used to drive *UAS-mahe* **(a, c )** or *UAS-CCT7-RNAi* **(b, c). (a-c and a’-c’)** Downregulation of *CCT7* enhanced the *mahe* induced eye roughness **(d-f)** *CCT7-RNAi* enhanced the *mahe* induced cell death. Scale bar in 50 µm **(a’-c’ and d-f)**. Genotypes **(a)** *w/+; +; GMR-GAL4,UAS-HA-mahe/+* **(b)** *w/+; +; CCT7-RNAi/GMR-GAL4* **(c)** *w/+; +; CCT7-RNAi/GMR-GAL4,UAS-HA-mahe.*


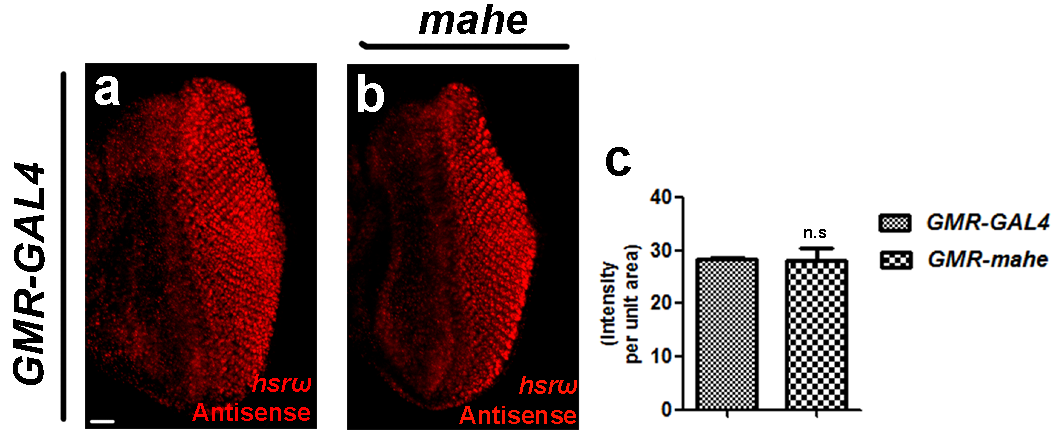


**Supplementary Figure S3: RNA-FISH was done to check the level of *hsrω* transcripts in eye antennal discs.** **(a)** FISH with *hsrω* specific antisense riboprobe in *GMR-GAL4* eye antennal disc. **(b)** Ectopic *mahe* expression shows same levels of *hsrω* transcript posterior to the morphogenetic furrow when compared to that of the control *GMR-GAL4*. **(c)** Graph represents intensity per unit area which shows no alteration in transcript level of *hsrω* in *mahe* overexpression and control tissues. Scale bar in 50μm **(a-b)**. Genotypes **(a)** *w/+; +; GMR-GAL4/+* **(b)** *w/+; +; GMR-GAL4, UAS-HA-mahe/UAS-HA-mahe.*
